# Validity and reliability of the Turkish version of the Holden Communication Scale

**DOI:** 10.1002/brb3.3223

**Published:** 2023-08-17

**Authors:** Özlem Bizpınar Munis, Mümüne Merve Parlak

**Affiliations:** ^1^ Department of Neurology Etlik City Hospital Ankara Turkey; ^2^ Department of Speech and Language Therapy, Faculty of Health Sciences Ankara Yıldırım Beyazıt University Ankara Turkey

**Keywords:** communication, dementia, mental status and dementia tests, psychometrics

## Abstract

**Introduction:**

The communication skills of individuals with dementia are affected even in the early stages of the condition. To date, there is no scale for the evaluation of communication abilities in Turkish‐speaking populations with dementia that can be used in clinical practice and research. The present study aimed to determine the validity and reliability of the Turkish version of the Holden Communication Scale (HCS‐TR).

**Materials and methods:**

The study was carried out with 141 participants (77 female and 64 male). Psychometric analyses were conducted to assess the internal consistency, construct and criterion validity, test–retest reliability, and inter‐rater reliability of the HCS‐TR. The Standardized Mini‐Mental State Examination (SMMSE) test was used for criterion validity. For the inter‐rater reliability of the scale, the two different caregivers of individuals with dementia were administered the scale separately at the same time. For test–retest reliability, 2 weeks later, the same caregivers who filled out the HCS‐TR the first time were administered to fill out the scale again. To test the validity of the scale, an item factor analysis was performed, and the correlations between the items and subsections were determined.

**Results:**

The factor loadings indicating the adequate contribution of the scale items to the relevant dimension were within the 0.700–0.831 range. There were positive relationships between all the items of the HCS‐TR, and there was a significant negative relationship (*r* = −.842) between HCS‐TR and MMSE. The corrected item–total correlation values were found to be within the .676–.794 range. Cronbach's alpha values for the HCS‐TR subsection and total scores in the first and second measurements were found to be in the range of .718–.944. There was no statistically significant difference (*p* = .709) between the mean total scores in the first and second measurements.

**Conclusion:**

The HCS‐TR is a valid and reliable tool that can be used for clinical and research purposes to assess the strengths and limitations of the communication skills of Turkish individuals with dementia.

## Graphical abstract

The communication skills of individuals with dementia are affected even in the early stages of the condition. To date, there is no Turkish communication scale for people with dementia that can be used for both clinical evaluation and research. This study demonstrates the validity and reliability of the Turkish version of the Holden Communication Scale.

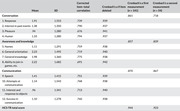

## 1 INTRODUCTION

Dementia is a syndrome characterized by a greater impairment of the cognitive function (i.e., the ability to process thought) than would be expected in normal old age (Parlak, Altan, et al., 2022; World Health Organization, 2017). Some of the cognitive processes, such as attention, memory, executive function, and language, are affected in individuals with dementia (Parlak, Tokgöz, et al., 2022; Parlak, Babademez, et al., 2022). Problems in these functions may affect verbal and nonverbal communication (Parlak & Köse, 2023; Parlak, Güç, et al., 2023). Even in the early stages of dementia, the communication skills of patients are affected, and this effect continues to increase as the severity of dementia increases (Parlak & Köse, 2023). Deficits in communication skills may vary in different types of dementia, but in the advanced stage, the language output of the patients is usually greatly reduced due to great cognitive destruction (Kim & Bayles, 2007).

Communication with people with dementia is important for person‐centered patient care. In addition, behavioral and psychological symptoms may be observed in individuals with dementia as a result of communication disorders (Selbæk et al., 2013). Therefore, it is necessary to identify communication deficits and strengths in individuals with dementia, especially those with moderate and severe cognitive impairment. Identifying the communication needs of individuals with dementia will enable caregivers and other communication partners to learn how to communicate with them. In addition, it is recommended that speech and language therapists work on the cognitive communication skills of individuals with dementia by conducting an assessment at an early stage. Communication assessment is important for drawing up a therapy program and guiding the course of therapy (Parlak & Köse, 2022; Parlak et al., 2023).

Various scales have been developed to evaluate different aspects of communication for individuals with dementia. However, most of the studies that developed these scales focused on the expression of agitation and aggression rather than communication skills (Egan et al., 2010; Strøm et al., 2016). Few dementia‐specific communication instruments focusing on communication skills have been developed. One of these is the Holden Communication Scale (HCS), a caregiver questionnaire originally developed to evaluate reality orientation and reminiscence therapy programs (Holden & Woods, 2015). It was revised in 2016, and its psychometric properties were determined (Strøm et al., 2016). HCS consists of 12 items assessing communication skills, and each item has 5 response options (0–4). It is divided into three sections: Conversation, Awareness and Knowledge, and Communication. There are four questions for each section. The first part (Conversation) observes parameters, such as initiative, interest, taste, and humor; the second part (Awareness and Knowledge) assesses environmental knowledge, such as names, orientation, general knowledge, and spontaneous activity; and the third part (Communication) assesses communication aspects, such as language, interest, reaction to objects, and success in communication (Strøm et al., 2016). The maximum score is 48 points, and the higher the score, the greater the difficulty in communication.

Unfortunately, to date, there is no communication scale specific to individuals with dementia in Turkey. The absence of a Turkish scale for both clinical assessment and research purposes in Turkish individuals with dementia constitutes a major gap. Thus, this study aimed to design a Turkish version of the HCS (HCS‐TR), examine its psychometric properties in a sample of individuals with dementia living with their families in Turkey, and determine its validity and reliability.

## 2 MATERIALS AND METHODS

The present study was performed at the Neurology Clinic of the University of Health Sciences, Dışkapı Training and Research Hospital, with the approval of the ethics committee (approval no. 132/04).

### 2.1 Study procedure

The present study consisted of two steps. The first step involved the translation and adaptation of HCS into Turkish, and the second step involved testing the validity and reliability of the translated scale.

The scale was translated into Turkish by the researchers after obtaining permission for such. HCS‐TR was then sent to three experts, whose opinions on the suitability of the translation and items were obtained. A pilot application of the translated scale was then conducted with 10 people, and their comprehension of the items was checked. The final version of the scale was decided based on the expert opinions and pilot application results. The scale was translated back into English by a translator who knew both Turkish and English but was not familiar with HCS‐TR. The English retranslation was then compared with the original English scale, and the expressions were found to be very similar.

Individuals with dementia who came to the dementia outpatient clinic within the last 2 years were identified in the system and informed about the study by phone call. Those who volunteered to participate in the study were called to the clinic for assessment. In the assessment, the Standardized Mini‐Mental State Examination (SMMSE) was performed by the neurologist, and the patient's dementia stage was determined using the clinical dementia rating scale. Without knowing the patients’ dementia stages or SMMSE test results, the speech and language therapist asked the patients’ caregivers to fill out HCS‐TR.

For the inter‐rater reliability of the scale, the two different caregivers of individuals with dementia were administered the scale separately at the same time. For test–retest reliability, 2 weeks later, the same caregivers who filled out HCS‐TR the first time were administered to fill out the scale again. To test the validity of the scale, an item factor analysis was performed, and the correlations between the items and subsections were determined. In addition, for the criterion validity of the HCS‐TR, the correlations between the SMMSE and HCS‐TR scores were analyzed for individuals with different dementia stages and for all the participants for the purpose of HCS‐TR construction validity.

### 2.2 Participants

A total of 128 individuals with intermediate and advanced dementia were included in the original version on the English HCS. The sample size for Likert‐type scales was calculated to be 5–20 participants per item (Pituch & Stevens, 2015), and because HCS‐TR is a 12‐item scale, at least 60–240 participants were required.

The names of 267 individuals with intermediate and advanced dementia were obtained from the hospital registration system. Of these, 63 were determined to have died. Individuals with dementia who were called for control were excluded from the study if they had severe pain or major depression, were in the palliative phase, were not expected to live for more than 6 months, or were not willing to participate in the study. After the exclusion criteria were met, 104 individuals with moderate‐advanced stage dementia and 37 individuals with mild‐stage dementia were included in the study. The study was conducted with a total of 141 participants.

### 2.3 Statistical analysis

The research data were analyzed using the SPSS 26 and AMOS 24 programs. Descriptive findings were given as numbers, percentages, means, and standard deviation values. The agreement between the two raters was evaluated through the Kappa analysis. The two caregivers’ HCS‐TR scores were grouped as follows in terms of communication: 0 (complete independence in communication), 1–12 (partial dependence in communication), 13–24 (moderate dependence in communication), 25–36 (advanced dependence in communication), and 37–48 (full dependence in communication). The construct validity of the scale was examined through confirmatory factor analysis using the AMOS program. The reliability of the scale was evaluated according to Cronbach's alpha coefficient, and a coefficient above .700 indicated that the scale was reliable (Gürbüz, 2019). The normality assumption of the variables was evaluated by considering the kurtosis and skewness values. The kurtosis and skewness values within ±1.5 showed a normal distribution (Tabach et al., 2013). Pearson's correlation coefficient was taken into consideration in the correlation analyses between variables. The difference between repeated measures was determined through paired *t*‐test analyses. The results of an inter‐item bias assessment were analyzed at a 95% confidence interval. A value of *p* < .05 was considered statistically significant in the evaluation of the analysis results.

## 3 RESULTS

Of the 141 study participants, 54.6% (*n* = 77) were female and 45.4% (*n* = 64) were male. The mean age of the participants was 76.16 ± 7.97, and the mean SMMSE score was 13.68 ± 8.822. Of the individuals with dementia, 40 (28.4%) were illiterate, 3 (2.1%) were literate, 79 (56.0%) were primary school graduates, 11 (7.8%) were secondary school graduates, 4 (2.8%) were high school graduates, 3 (2.1%) were university graduates, and 1 (0.7%) was postgraduate.

In the confirmatory factor analysis conducted to evaluate the construct validity of HCS‐TR, the factor loadings were found to be within the 0.700–0.831 range, which were statistically significant (*p* < .001; Table 1). The fit index values related to the analysis are given in Table 2.

**TABLE 1:** Confirmatory factor analysis (*n* = 141).

Parameter		Estimate	Lower	Upper	*p*
**Item 1**	Conversation	0.750	0.676	0.816	**.000**
**Item 2**	Conversation	0.799	0.716	0.870	**.000**
**Item 3**	Conversation	0.700	0.591	0.787	**.000**
**Item 4**	Conversation	0.821	0.743	0.886	**.000**
**Item 5**	Awareness and knowledge	0.806	0.726	0.869	**.000**
**Item 6**	Awareness and knowledge	0.792	0.720	0.856	**.000**
**Item 7**	Awareness and knowledge	0.831	0.758	0.891	**.000**
**Item 8**	Awareness and knowledge	0.756	0.673	0.831	**.000**
**Item 9**	Communication	0.799	0.702	0.875	**.000**
**Item 10**	Communication	0.814	0.735	0.878	**.000**
**Item 11**	Communication	0.753	0.657	0.829	**.000**
**Item 12**	Communication	0.807	0.716	0.878	**.000**

In bold, *p*‐value < 0.05 considered statistically significant.

**TABLE 2:** Concordance index (*n* = 141).

Concordance index	Value	Good compliance	Acceptable compliance
***χ* ^2^**	75.557		
**SD**	49		
***χ* ^2^/SD**	1.541	≤3	≤5
**GFI**	0.995	0.95 ≤ GFI ≤ 1.00	0.90 ≤ GFI < 0.95
**AGFI**	0.992	0.90 ≤ AGFI ≤ 1.00	0.85 ≤ AGFI < 0.90
**NFI**	0.994	0.95 ≤ NFI ≤ 1.00	0.90 ≤ NFI < 0.95
**RFI**	0.992	0.95 ≤ RFI ≤ 1.00	0.90 ≤RFI < 0.95
**SRMR**	0.042	0 < SRMR ≤ 0.05	0.05 < SRMR ≤ 0.10

Abbreviations: AGFI, adjustment goodness of fit index; GFI, goodness of fit index; NFI, normed fit index; RFI, relative fit index; SD, standard deviation; *χ* ^2^, Chi‐squared; SRMR, standardized root mean square residual.

With regard to the correlations between the HCS‐TR items, all the items were found to have a positive relationship. The highest relationship (.746) was between “10, communication trials (attempts)” and “12, success in communication,” whereas the lowest relationship (.429) was between “3, joy (satisfaction)” and “6, general orientation” (Table 3).

**TABLE 3:** Correlation matrix for the 12 items in Turkish version of the Holden Communication Scale (HCS‐TR) (*n* = 141).

	Item 1	Item 2	Item 3	Item 4	Item 5	Item 6	Item 7	Item 8	Item 9	Item 10	Item 11	Item 12
Item 1	1.000											
Item 2	0.735	1.000										
Item 3	0.514	0.508	1.000									
Item 4	0.623	0.663	0.626	1.000								
Item 5	0.504	0.649	0.526	0.705	1.000							
Item 6	0.575	0.609	0.429	0.655	0.672	1.000						
Item 7	0.570	0.631	0.487	0.694	0.705	0.744	1.000					
Item 8	0.604	0.536	0.520	0.576	0.505	0.477	0.602	1.000				
Item 9	0.632	0.689	0.502	0.579	0.590	0.547	0.584	0.616	1.000			
Item 10	0.560	0.623	0.659	0.602	0.577	0.547	0.590	0.569	0.618	1.000		
Item 11	0.488	0.565	0.548	0.587	0.605	0.518	0.564	0.542	0.567	0.604	1.000	
Item 12	0.624	0.633	0.615	0.587	0.591	0.530	0.548	0.495	0.590	0.746	0.657	1.000

The correlation analysis conducted to evaluate criterion validity showed that there was a significant negative relationship (*r* = −.842) between the HCS‐TR and SMMSE scores. Correlation analyses were also performed according to the SMMSE score groups, and negative correlations were found between the HCS‐TR scores and the low, medium, and high SMMSE score groups (*r* = −.656; *r* = −.445; *r* = −.407, respectively; Table 4).

**TABLE 4:** Turkish version of the Holden Communication Scale (HCS‐TR) and Standardized Mini‐Mental State Examination (SMMSE) correlation analysis results (*n* = 141).

	Low SMMSE score 0–10 (*n* = 58)	Medium SMMSE score 11–20 (*n* = 46)	High SMMSE score 21–30 (*n* = 37)
**Conversation**			
1. Response	−.105	−.446^**^	−.259
2. Interest in past events	−.489^**^	−.408^**^	−.179
3. Pleasure	−.375^**^	−.103	−.014
4. Humor	−.589^**^	−.193	−.342^*^
**Awareness and knowledge**			
5. Names	−.597^**^	.012	−.234
6. General orientation	−.454^**^	−.288	−.380^*^
7. General knowledge	−.634^**^	−.325^*^	−.503^**^
8. Ability to join in games, etc.	−.227	−.211	−.341^*^
**Communication**			
9. Speech	−.379^**^	−.372^*^	−.352^*^
10. Attempts at communication	−.335^*^	−.162	−.067
11. Interest and response to objects	−.585^**^	−.152	−.074
12. Success in communication	−.508^**^	−.317^*^	−.124
**HCS‐TR total score**	−.656^**^	−.445^**^	−.407^*^

* *p* < .05.

** *p* < .01.

The corrected item–total correlation findings and Cronbach's alpha values for the first‐ and second‐measurement HCS‐TR items are given in Table 5. According to these findings, the corrected item–total correlation values were within the .676–.794 range. Cronbach's alpha values for the HCS‐TR subsection and total scores in the first and second measurements were found to be within the .718–.944 range.

**TABLE 5:** Turkish version of the Holden Communication Scale (HCS‐TR) items corrected item–total correlation and Cronbach's alpha results (*n* = 141).

	Mean	SD	Corrected item–total correlation	Cronbach's *α* if item deleted	Cronbach's *α* first measurement (*n* = 141)	Cronbach's *α* second measurement (*n* = 54)
**Conversation**					.861	.718
1. Response	1.91	1.553	.739	.939		
2. Interest in past events	1.38	1.350	.790	.937		
3. Pleasure	.94	1.280	.676	.941		
4. Humor	1.33	1.280	.794	.937		
**Awareness and knowledge**					.857	.839
5. Names	1.11	1.291	.759	.938		
6. General orientation	2.23	1.495	.719	.940		
7. General knowledge	1.98	1.360	.775	.938		
8. Ability to join in games, etc.	2.22	1.682	.691	.942		
**Communication**					.870	.867
9. Speech	1.41	1.415	.751	.939		
10. Attempts at communication	1.14	1.543	.768	.938		
11. Interest and response to objects	.96	1.341	.713	.940		
12. Success in communication	1.10	1.278	.760	.938		
**HCS‐TR total score**					.944	.923

It was determined through a paired *t*‐test that there was no statistically significant difference (*p* = .709) between the mean HCS‐TR total score in the first measurement (18.74 ± 12.579) and that in the second measurement (19.12 ± 12.453). When bias was evaluated in terms of items, it was observed that there was a small bias only in the “first response” item (*p* = .032), and there was no bias in the other items or the total score at a 95% confidence interval (Table 6). In addition, the correlation test between the total scores of the first and second measurements showed a significant positive correlation (*r* = .817).

**TABLE 6:** Results of difference analysis between measurements (*n* = 54).

	First measurement		Second measurement			95% limits of agreement		
	Mean	SD	Mean	SD	Bias	Lower	Upper	*p*
**Conversation**								
1. Response	1.91	1.496	1.44	1.313	0.002	0.093	0.889	.032
2. Interest in past events	1.31	1.343	1.28	1.309	0.001	–0.277	0.370	.816
3. Pleasure	0.83	1.285	1.04	1.331	0.006	–0.611	0.203	.338
4. Humor	1.56	1.369	1.61	1.366	–0.004	–0.444	0.333	.792
**Awareness and knowledge**								
5. Names	1.11	1.341	1.20	1.188	0.002	–0.351	0.167	.510
6. General orientation	2.39	1.379	2.57	1.409	–0.003	–0.444	0.074	.173
7. General knowledge	2.19	1.245	2.30	1.341	0.000	–0.389	0.167	.462
8. Ability to join in games, etc.	2.41	1.548	2.61	1.571	0.002	–0.611	0.241	.370
**Communication**								
9. Speech	1.63	1.405	1.44	1.633	0.003	–0.204	0.593	.371
10. Attempts at communication	1.17	1.502	1.39	1.676	–0.019	–0.685	0.185	.291
11. Interest and response to objects	1.07	1.385	1.15	1.497	–0.009	–0.444	0.278	.730
12. Success in communication	1.17	1.240	1.09	1.186	–0.008	–0.278	0.444	.693
**HCS‐TR total score**	18.74	12.579	19.12	12.453	–0.028	–2.499	1.499	.709

Abbreviation: HCS‐TR, Turkish version of the Holden Communication Scale.

## 4 DISCUSSION

The present study was the first to design a Turkish version of the English HCS for individuals with dementia living with their families in Turkey and to test the validity and reliability of the scale. The present study was conducted with 141 participants. In addition, not only individuals with moderate‐ and advanced‐stage dementia but also those with mild‐stage dementia were included in the study because it had been previously reported that the communication skills of individuals with mild‐stage dementia could also be affected to different degrees.

For construct validity, factor loadings above 0.500 are desirable (Gürbüz, 2019). The factor loadings of the HCS‐TR items were in the 0.700–0.831 range. This shows that the scale items contribute to the relevant dimensions at a sufficient level; that is, they are in the appropriate subsection of the questions of the HCS‐TR. In the confirmatory factor analysis, the Chi‐squared/Standard deviation (*χ* ^2^/SD) value was 1.541. When this value is equal to or less than 3, the model is considered to be a good fit (Schermelleh‐Engel et al., 2003), and therefore, our results showed that the model used in the study had a good fit. Goodness of fit index, adjustment goodness of fit index, normed fit index, and relative fit index values of .95 and above are regarded as excellent fit values; thus, HCS‐TR was found to have an excellent goodness of fit (Schermelleh‐Engel et al., 2003). These results show that the 12‐item HCS‐TR with 3 basic subdimensions has construct validity.

The internal consistency correlations between the HCS‐TR items were all positive, within .429–.746. All the items had correlations of .504 or above, except for items 11 and 1. Thus, all the items measured the same underlying aspect of communication, which correlated with the total score. High and positive item–total correlations indicate that the items in the measurement tool have similar behaviors and that the internal consistency of the scale is high. The item–total correlations of the items in the measurement tool should be .30 and above, and the items with these values are good items (Büyüköztürk, 2018). The corrected item–total correlation values of HCS‐TR were found to be within the .676–.794 range. This result is similar to that obtained by Strøm et al. (2016). These results show that all the items in HCS‐TR are reliable.

Cronbach's alpha internal consistency reliability is mostly used by researchers among the reliability determination methods in the measurement tool development stages (Acar Güvendir & Özer Özkan, 2015). Cronbach's alpha value of the HCS‐TR total score was .944 in the first measurement and .923 in the second measurement. This shows that HCS‐TR has excellent reliability according to the evaluation criteria specified by George and Mallery (2003). For the subdimensions, it can be said that there is a good level of subdimension reliability because Cronbach's alpha value ranges from .857 to .87. Cronbach's alpha value of .70 and above is considered sufficient for the reliability of a measurement tool, and a measurement tool that meets this value is considered reliable (Büyüköztürk, 2018). Therefore, our findings show that HCS‐TR is a reliable scale.

HCS‐TR was readministered 2 weeks later to determine its test–retest reliability, and it was determined that there was a significant positive correlation between the total scores (*r* = .935). Strøm et al. took the retest period of 1 week and found a significant bias in the item of participation in meaningful games in the retest measurement. In the present study, a significant bias was found in the response item (*p* = .032), and it was observed that the first item decreased from 1.91 to 1.44 in the second measurement. This result could have been due to the fact that some patients in the advanced dementia stage changed their response levels during the 2‐week period. However, the findings indicate that HCS‐TR has good test–retest reliability, and it can be concluded that caregivers of individuals with dementia consistently assess such individuals’ communication skills. When the inter‐rater reliability of HCS‐TR was analyzed by applying it to two different caregivers, the alpha values for HCS‐TR were found to be weakly correlated according to the values defined by Viera and Garrett (2005) and moderately correlated according to the values defined by Landis and Koch (1977). These were the results of 32 caregivers. These results could have been influenced by the difference in the time that the two caregivers spent with the patients because the caregiver who spent more time with the patient likely had more information about their conditions. In the case of alternating caregiving, depending on the underlying etiology of dementia, there may be a change in communication skills during the progression, and this may cause a difference between the scores given by the last and previous caregivers. For this reason, the duration and time intervals of caregivers’ interactions with individuals with dementia may also be considered. In addition, it is known that the fewer the categories, the larger the Kappa value (Sim & Wright, 2005). In the present study, the Kappa score was calculated by analyzing the HCS‐TR scores in five categories, and the results could have changed by reducing the number of categories.

High HCS‐TR scores indicate serious communication problems. In the present study, a significant negative correlation (*r* = −.842) was found between the HCS‐TR and SMMSE scores. This result showed that the communication disorders increased as the cognitive impairment increased. Therefore, the clinical validity of HCS‐TR can be considered high. In addition, the correlation results showed that the rate of increase in the HCS‐TR total score was higher as the SMMSE scores decreased. This result shows that HCS‐TR is a sensitive tool that can be used to determine communication disorders and their severity in Turkish‐speaking individuals with dementia, and that the rate of deterioration of communication skills may be much faster and higher as the stage of the condition progresses.

The correlation results of the HCS‐TR and SMMSE scores in individuals with different stages of dementia were analyzed, and it was found that the correlation was highest in individuals with advanced dementia. Although statistically significant, the correlation coefficients were lower in the moderate and mild stages (*r* = −.656; *r* = −.445; *r* = −.407). However, Strøm et al. found a correlation as low as *r* = −.06 between the HCS and SMMSE scores of individuals with mild‐stage dementia. According to Strøm et al., the fact that patients with moderate‐stage dementia showed a higher correlation means that HCS‐TR is more valid for the moderate dementia stage than for the other stages. In addition, the present study showed that HCS‐TR, which was basically designed for people with moderate and advanced cognitive impairment, could also be used for participants with mild cognitive impairment. Thus, the HCS‐TR may be used as an additional source of reference to determine the level of cognitive impairment in individuals living with dementia, evaluate their communication skills, and identify the areas where they need support.

In the present study, the validity and reliability of HCS‐TR were tested only in individuals with dementia. To date, however, there is no Turkish communication assessment scale for other cognitive communication disorders, such as traumatic brain injury and right hemisphere injury. Thus, in future studies, the validity and reliability of the scale for different cognitive communication disorders, such as traumatic brain injury, can be tested.

## 5 CONCLUSION

HCS‐TR is a valid and reliable tool that can be used for clinical and research purposes to assess the strengths and limitations of the communication skills of Turkish individuals with dementia. The analysis results show that the scale has high internal consistency and test–retest reliability. In addition, the correlations between cognitive function and communication skills increased as the severity of dementia increased. HCS‐TR is an easy‐to‐use tool and can be used in all stages of dementia, including the mild stages. It can provide important information about communication skills for drawing up a therapy program by assessing people with dementia and directing the course of therapy.

## AUTHOR CONTRIBUTIONS

**Mümüne Merve Parlak**: Conceptualization; data collection; data analysis; writing of the manuscript. **Özlem Bizpınar Munis**: Conceptualization; data collection and critical editing of the manuscript. All the authors approved the final version of the manuscript.

## CONFLICT OF INTEREST STATEMENT

The authors have no conflicts of interest to declare.

### PEER REVIEW

The peer review history for this article is available at https://publons.com/publon/10.1002/brb3.3223.

## Data Availability

All data generated or analyzed during this study are included in this article. Further enquiries can be directed to the corresponding author.
